# The complete mitochondrial genome of *Philus pallescens* Bates, 1866 (Coleoptera: Vesperidae) and its phylogenetic implications

**DOI:** 10.1080/23802359.2022.2034544

**Published:** 2022-03-06

**Authors:** Liang-Jong Wang, Li Lin, Chia-Hsuan Sung, Ming-Ying Lin, Meng-Hao Hsu

**Affiliations:** a Division of Forest Protection, Taiwan Forestry Research Institute, Taipei, Taiwan; b Crop Environment Section, Hualien District Agricultural Research and Extension Station, Hualien, Taiwan; c Planning and Information Division, Fisheries Research Institute, Keelung, Taiwan; d Department of Plant Medicine, National Chiayi University, Chiayi City, Taiwan

**Keywords:** Mitochondrial genome, *Philus pallescens*, Coleoptera, Vesperidae, next-generation sequencing

## Abstract

We sequenced and assembled the complete mitochondrial genome of *Philus pallescens* from Madou, Tainan County, Taiwan. The complete mitogenome of *P. pallescens* is 15,750 bp long, and contains 13 protein-coding, 22 tRNA and two rDNA genes. Nucleotide compositions of the mitogenome of *P. pallescens* are A: 38.08%, T: 32.25%, C: 18.67%, and G: 11.00%. The AT and GC skewness of the mitogenome sequence were 0.0828 and −0.25845 respectively, showing the genome composition skewed toward adenine and cytosine. The phylogenetic position of Chrysomelidae is sister to all the other families in the superfamily Chrysomeloidea. The results indicate that Chrysomeloidea Cerambycidae is not a monophyletic group. *Philus* is phylogenetically close to Spiniphilus. Vesperidae is monophyletic and sister to Disteniidae. Mitogenomic data from this study will provide useful information for further studies on the population genetics, speciation, and pest management of *P. pallescens*.

Four families, the Vesperidae Mulsant, 1839, Oxypeltidae Lacordaire, 1868, Disteniidae Thomson, Disteniidae J. Thomson,1861 and Cerambycidae Latreille, 1802 s.s., comprise Longicornia or so-called longhorn beetles in the current classification (Švácha and Lawrence 2014). The family Cerambycidae s.s. includes the subfamilies Lamiinae, Cerambycinae, Lepturinae, Prioninae, Dorcasominae, Parandrinae, Spondylidinae, and Necydalinae. Vesperidae comprises 17 described genera with nearly 80 species in the world (Švácha and Lawrence 2014). The genus *Philus saunders*, 1853 comprises 10 species and distributes in Asia (Tavakilian and Chevillotte 2021). *Philus pallescens* was described based on the material collected by R. Swinhoe from Taiwan (Bates 1866). The host plant of *P. pallescens* is *Saccharum officinarum*, and the larvae feed on its roots and the lower part of the stem (Duffy 1968). *P. pallescens* is distributed in Taiwan and south-eastern China. Its flight period is mainly from May to June in Taiwan. This is the first report of its complete mitochondrial sequences.

The single specimen of *P. pallescens* in this study was collected by use of a light trap in Madou, Tainan County, Taiwan (23°09'15.2"N 120°14'43.3"E) in May 2016. Total genomic DNA was extracted from the legs of the adult using a QuickExtract™ DNA Extraction Solution kit (Epicentre, Madison, WI, USA) following the supplier’s instructions. The voucher specimen (accession number: Ppa2016W003) and its genomic DNA (accession number: Ppa2016WGDNA003) were deposited in the Lab. of Forest Insects and Systematic Entomology, Taiwan Forestry Research Institute, Taipei, Taiwan (contact person: L. J. Wang, ljwang23@ms17.hinet.net). The voucher specimen and other specimens collected in the same site were identified to species level by L. J. Wang based on the reference (Bates 1866; Yu et al. 2002). The complete mitogenome of *P. pallescens* was sequenced using the next-generation sequencing method (Illumina MiSeq, San Diego, CA). A total of 1.06 Gb next-generation sequencing paired-end reads were used to assemble the complete mitogenome sequence (Hahn et al. 2013). The CLC Genomics Workbench ver.12.0.02 (QIAGEN, Hilden, Germany) was used for sequence quality analysis, data-trimming, and de novo assembly by default setting. The assembled contigs were then used to BLAST against the mitogenomes of the species in the family Versperidae *Philus antennatus* (NCBI acc. MN473120), *Spiniphilus spinicornis* (NCBI acc. MN420470) and *Mantitheus pekinensis* (NCBI acc. MN473092) to identify the targeted contig with the highest BLAST score and lowest e-value as the mitogenome of *P. pallescens*. The locations of the protein-coding genes, ribosomal RNAs (rRNAs), and transfer RNAs (tRNAs) were predicted by using MITOS Web Server (Bernt et al. 2013) and identified by alignment with other mitogenomes of Versperidae species. The AT and GC skews were calculated according to the following formulae: AT skew=(A–T)/(A + T) and GC skew=(G–C)/(G + C) (Perna and Kocher 1995). The phylogenetic reconstruction based on Maximum likelihood (ML) analyses was performed using the GTRGAMMA model implemented in RAxML v.8.1.17 (Stamatakis 2014). Nodal support confidence was estimated using a fast bootstrapping analysis with 1000 replicates in RAxML with the model GTRCAT. The phylogenetic analyses consist of 37 mitochondrial genomes within the superfamily Chrysomeloidea and *Curculio davidi* (Curculionidae) was selected as the outgroup. The concept of family level followed Švácha and Lawrence (2014).

The complete mitogenome of *P. pallescens* is 15,750 bp in length (GenBank Accession No. MZ747394), including 13 protein-coding genes, two rRNA genes, 22 tRNA genes and one control region. The total nucleotide compositions of the *P. pallescens* mitogenome are A: 38.08%, T: 32.25%, C: 18.67%, and G: 11.00%. The AT and GC skews of the mitogenome sequence are 0.0828 and −0.25845, showing the genome composition skewed toward adenine and cytosine. The gene rearrangement of the mitogenome in *P. pallescens* is identical to the ancestral inferred insect type (Cameron 2014). The phylogenetic tree was reconstructed based on 13 mitochondrial protein-coding genes (Figure 1). Bootstrap values are shown at the branch nodes. The phylogenetic position of Chrysomelidae (*Callosobruchus analis* (KY856745)) is sister to all the other families in the superfamily Chrysomeloidea. Two major clades are presented in the present result, one clade comprises (((Cerambycinae + Prioninae)+Dorcasominae)+ (Disteniidae + Vesperidae))+ (Orsodacnidae + Oxypeltidae))), and the other clade comprises ((Lepturinae+(Lamiinae+ (Aseminae + Spondylidinae)). This result is consistent with the molecular phylogenetic result of Nie et al. (2021) indicating that Cerambycidae is not monophyletic. The clade including *P*. *pallescens* and *Philus antennatus* received absolute support (100%). *Philus* is phylogenetically close to *Spiniphilus*. Vesperidae is monophyletic and sister to Disteniidae, consistent with the results of Nie et al. (2021). Mitogenomic data from this study will provide useful information for further studies on the population genetics, speciation, and pest management of *P. pallescens*.

**Figure 1.**
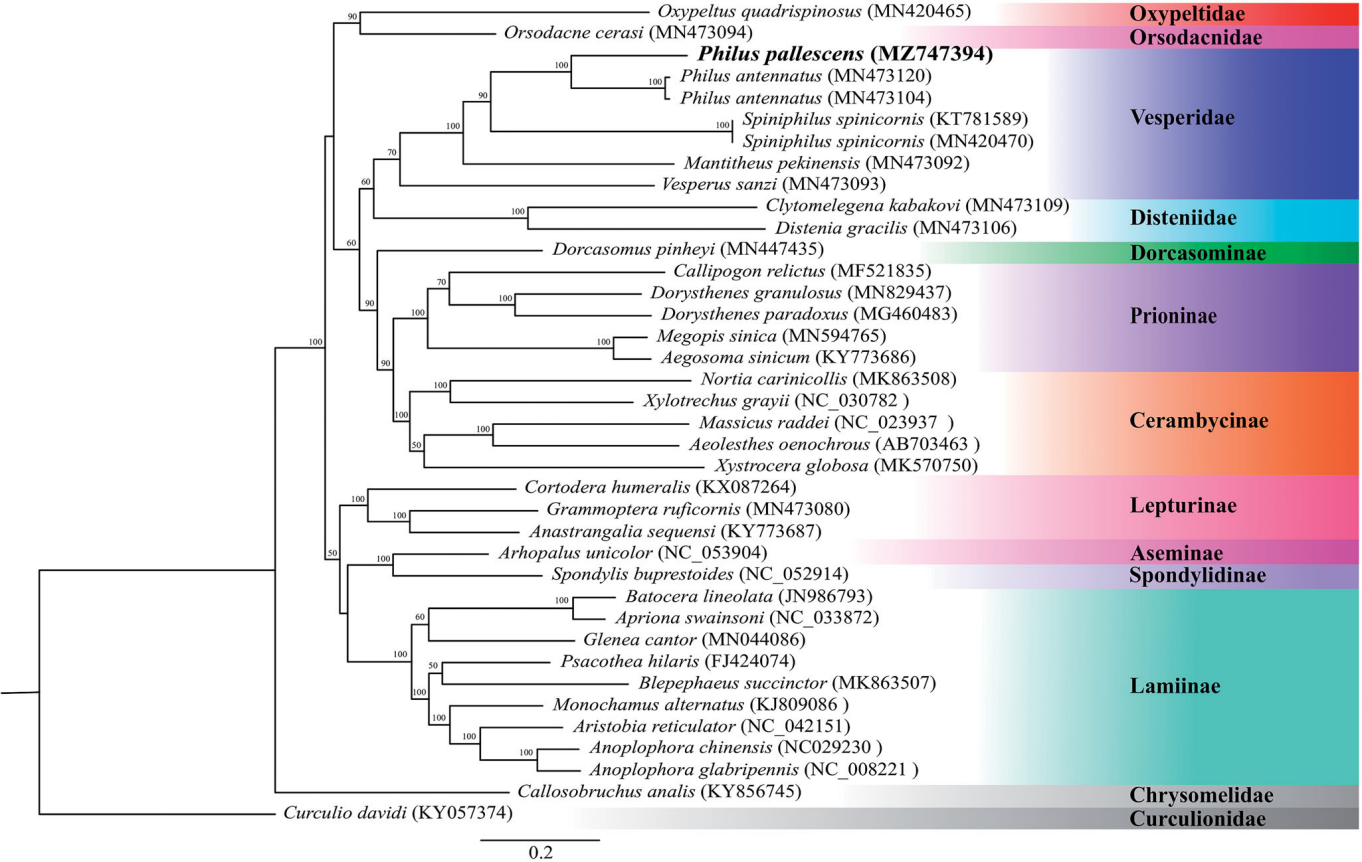
Phylogenetic tree of 36 species of Chrysomeloidea including *Philus pallescens* (in this study, MZ747394) and 1 outgroup based on the sequence of mitochondrial 13 protein-coding genes. The tree was reconstructed under the GTRGAMMA model implemented in RAxML v.8.1.17 (Stamatakis 2014). Nodal support confidence was estimated using a fast bootstrapping analysis with 1000 replicates in RAxML with the model GTRCAT.

## Data Availability

The genome sequence data that support the findings of this study are openly available in GenBank of NCBI (National Center for Biotechnology Information) at [https://www.ncbi.nlm.nih.gov] under the accession no. MZ747394. The associated BioProject, SRA, and Bio-Sample numbers are PRJNA755352, SRR15532969, and SAMN20821259 respectively.
